# Effect of Integrated Care on Patients With Atrial Fibrillation: A Systematic Review of Randomized Controlled Trials

**DOI:** 10.3389/fcvm.2022.904090

**Published:** 2022-05-17

**Authors:** Yi Li, Wenjing Zhao, Jun Huang, Murui Zheng, Peng Hu, Jiahai Lu, Hai Deng, Xudong Liu

**Affiliations:** ^1^Department of Epidemiology, School of Public Health, Sun Yat-sen University, Guangzhou, China; ^2^School of Public Health, Guangdong Pharmaceutical University, Guangzhou, China; ^3^School of Public Health and Emergency Management, Southern University of Science and Technology, Shenzhen, China; ^4^Department of Geriatrics, Institute of Geriatrics, Guangdong Provincial People’s Hospital, Guangdong Academy of Medical Science, Guangzhou, China; ^5^Guangzhou Center for Disease Control and Prevention, Guangzhou, China; ^6^Department of Cardiology, Guangdong Cardiovascular Institute, Guangdong Provincial People’s Hospital, Guangdong Academy of Medical Science, Guangzhou, China

**Keywords:** atrial fibrillation, integrated care, prognosis, meta-analysis, systematic review

## Abstract

**Aims:**

The integrated management was evidenced to improve the hospitalization and its associated complications in patients with atrial fibrillation (AF), but the strategies of integrated care varied and results were inconsistent. This systematic review and meta-analysis aimed to evaluate the effect of integrated care on AF-related outcomes with comparison with usual care.

**Methods:**

PubMed, Embase, and Web of Science were searched for articles published until 10th January 2022. Eligible studies were randomized controlled trials to study the effect of integrated care on AF-related outcomes. Meta-analysis with a random-effect model was used to calculate risk ratio (RR) and 95% confidence interval (CI) by comparing the integrated care with usual care.

**Results:**

A total of five studies with 6,486 AF patients were selected. By synthesizing available data, integrated care effectively reduced the risk of all-cause mortality (RR = 0.54, 95% CI = 0.42–0.69), cardiovascular hospitalization (RR = 0.72, 95% CI = 0.55–0.94), and cardiovascular mortality (RR = 0.52, 95% CI = 0.36–0.78) when compared with usual care; however, there was no superior effect on preventing AF-related hospitalization (RR = 0.86, 95% CI = 0.72–1.02), cerebrovascular events (RR = 1.13, 95% CI = 0.75–1.70), and major bleeding (RR = 1.29, 95% CI = 0.86–1.94) when comparing integrated care with usual care.

**Conclusion:**

Integrated care can reduce the risk of all-cause mortality, cardiovascular mortality, and cardiovascular hospitalizations in AF patients compared with usual care, while the benefit was not observed in other outcomes.

## Introduction

Atrial fibrillation (AF) is the most common cardiac arrhythmia and the leading cause of hospitalization for arrhythmias. Studies have shown that hospitalization and its associated complications in AF patients can be significantly improved with care through integrated management (1). Integrated care is a patient-oriented approach, providing patients with personalized care and optimized treatment by interdisciplinary teams (2, 3). In the model of integrated care, the treatment of AF varied according to the patient’s condition and the emergence of new therapies (3). Integrated AF care can significantly reduce the treatment burden of patients and enhance patients’ compliance to treatment (4).

However, the current evidence did not yield a consistent conclusion on the AF prognosis by the implementation of integrated care (5–7). In addition, more trials (RCT) (8–10) have been reported and the newly released guidelines for the diagnosis and treatment of AF placed further emphasis on the participation of patients and the involvement of families/caregivers (2). All these prompted us to update the review and to provide new synthetic evidence. Therefore, this systematic review and meta-analysis by synthesizing the existing randomized control trials (RCTs) intended to evaluate the impact of integrated care on the prognosis of AF patients, compared with traditional usual care.

## Methods

### Search Strategy and Study Selection

This study was a systematic review and meta-analysis and was conducted according to the PRISMA statement (11). PubMed, Embase, and Web of Science were searched independently by two reviewers (YL and WZ) for articles published until 10th January 2022, with search strategy of “atrial fibrillation” AND (delivery of health care, integrated [MeSH Terms] OR “integrated health care” OR “integrated care” OR “nurse-led care” OR “Interdisciplinary Communication” OR “Interdisciplinary Communications” OR multidisciplinary OR “outpatient” OR “ambulatory care” OR nursing OR “ABC pathway” OR “ABC care”) AND (“mortality” OR “death” OR “all-cause mortality” OR “hospitalization” OR “hospital admissions”, OR “stroke” OR “major bleeding” OR “adherence to guidelines” OR “quality and outcomes” OR “multimorbidity” OR “anticoagulation”). The detail of the search strategy was displayed in Supplementary Table 1. The group discussion with the third researcher (XL) was carried out to resolve the disagreement. The references of articles were also tracked to find potential articles.

Inclusion criteria were as follows: the effect of integrated care on the potential outcome of AF patients, with a comparison to usual care, was reported or can be calculated; study design was the RCT; AF-related outcomes including all-cause mortality, cardiovascular hospitalizations, AF-related hospitalizations, cerebrovascular events, cardiovascular mortality, and major bleeding were reported. Observational studies, animal studies, reviews, editorials, letters, non-randomized controlled trials, abstracts, and studies of lacking data to manifest the effect of integrated care on AF-related outcomes were excluded.

### Data Extraction and the Risk of Bias Assessment

The information extracted from each trial included the first author, year of publication, country, number of participants, the proportion of women, mean or median of age, follow-up years, CHADS_2_-VASc score, AF-related outcomes, and intervention strategies for both the integrated care group and the usual care group. The risk of bias for each study was evaluated by using the Cochrane tool (12).

### Statistical Analyses

A meta-analysis was conducted to evaluate the pooled effect of integrated care on the development of AF-related outcomes, including all-cause mortality, cardiovascular mortality, cardiovascular hospitalizations, AF-related hospitalizations, major bleeding events, and cerebrovascular events. The count of events was extracted from each study, and the pooled effect displayed as risk ratio (RR) and 95% confidence interval (CI) was calculated using a random-effects model (Mantel-Haenszel approach). The heterogeneity among the studies was assessed by using I^2^ statistic and Q-test. I^2^ value of more than 50% or P-value from Q-test of less than 0.05 suggested significant heterogeneity (13). A funnel plot was used to visually assess the publication bias. The sensitivity analysis was done by excluding one study at a time. All analyses were performed using Review Manager (Version 5.3.), and a two-tailed *P* value less than 0.05 was deemed to be significant.

## Results

**FIGURE 1:**
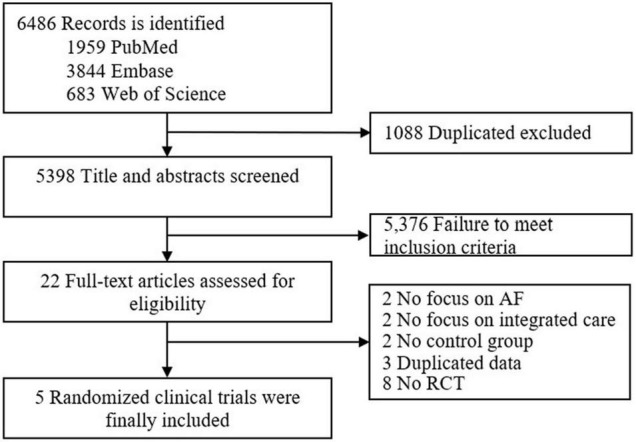
Flowchart for publication selection.

The procedure of studies’ selection is shown in Figure 1. A total of 6,486 articles were systematically identified from PubMed, Embase, and Web of Science. After removing 1,088 duplicated records, 5,398 articles were left for the title and abstract screening. Among the 22 articles for further full-text reviewing, 17 articles were excluded for non-randomized controlled trials, not focusing on AF or integrated care, having no control group, and duplicate data (Supplementary Table 2). Finally, five studies (6–10) were included in this study. The assessment of the risk of bias is shown in Supplementary Figure 1. All five included trials were at low risk. However, in three trials the risk of selection bias due to allocation concealments was unclear and in five trials the risk of performance bias due to blinding of participants and personnel was unclear.

The detailed characteristic of each study is presented in Table 1. A total of 6,986 adult participants were included in five selected studies, and among them about 38–49% were female. The mean or median age ranged from 64 to 77 years old; the follow-up period ranged from 0.8 to 3.08 years. Three studies were conducted in Netherlands (7–9), and two in Australia (6) and China respectively (10). Three focused on tertiary hospital care setting (6–8) and two on primary care setting (9, 10). Three studies (8–10) included patient/family involvement in their integrated care approach, but the other two did not (6, 7). Two studies reported the health-related quality of life (6, 9), while the other three did not (7, 8, 10).

**TABLE 1:** Characteristics of included studies.

Author, Publication year, Country, Study design	Setting	Study period	Total participants	Proportion of women (%)	Mean (SD) or median of age (years)	Follow-up years	CHA2DS_2_-VASc score	Primary outcome	Second outcome
Stewart et al. (6) Australia, pragmatic multicenter, randomized controlled trial	Hospital care	2010.06.02–2014.03.31	335	48	71.5 (12)	2.51	3.7 ± 1.8 in IG vs. 3.6 ± 1.9 in CG	Death and unplanned readmission (both all-cause)	Unplanned, CV specific, and all-cause readmission and length of hospital stay
Hendriks et al. (7) Netherland, One-center Randomized clinical trial	Hospital care	2007.01–2009.12	712	41.3	67 (13)	1.83	≥1 score: 127 (35.7%) patients in IG vs 126 (35.4%) patients in GG	CV hospitalization and death	NA
Wijtvliet et al. (8) Netherlands, Multi-center Randomized clinical trial	Hospital care	2012.12–2018.10	1,375	44	64 (10)	3.08	≥2 score: 387 (58%) patients in IG vs 379 (56%) patients in GG	CV hospitalization and death	The level of implementation of care
van den Dries et al. (9) Netherlands, Cluster randomized pragmatic non-inferiority trial	Primary care	2015.10–2019.03	1,240	49	77 ^†^	≥2	NA	All-cause mortality	CV and non-CV mortality, CV and non-CV hospitalization, MACE, stroke, major bleeding, CRNMB, HrQoL, and cost-effectiveness
Guo et al. (10) China, Cluster randomized controlled trial	Primary care	2017.09–2019.08	3,324	38	68.5 (15)	0.8	3 (2–4)	Stroke/thromboembolism, all-cause death, and rehospitalization.	Event rates of the primary endpoint, and the change in the proportion of patients’ anticoagulation

*CV, cardiovascular; ED, emergency department; AF, atrial fibrillation, MACE, major adverse cardiac events: CRNMB, clinically relevant non-major bleeding; HrQoL, health-related quality of life; IG, intervention group; CG, control group.*

*^†^Median of age.*

Four studies (6, 7, 9, 10) reported the outcome of all-cause mortality, three (6–8) reported AF-related hospitalizations, three (7–9) reported major bleeding, four (7–10) reported cardiovascular mortality and all five studies (6–10) reported cardiovascular hospitalizations and cerebrovascular events. All included studies used usual care as a reference when estimating the effect of integrated care. The detailed intervention strategies of integrated care and usual care for each study are shown in Supplementary Table 3. The essential elements of the integrated AF management strategy adopted in each study are shown in Supplementary Table 4. All five studies (6–10) considered four elements, including optimized stroke prevention, symptom control with rate or rhythm control, patient education/self-management, structured follow-up and clear communication between primary and secondary care; four studies (7–10) considered multidisciplinary team approach; three studies (6, 8, 10) considered two elements of management of cardiovascular risk factors/comorbidities, and strategies to promote medication adherence; two studies (7, 8) considered psychosocial management, two studies considered healthcare professional education; only one study (9) considered lifestyle modification.

**FIGURE 2:**
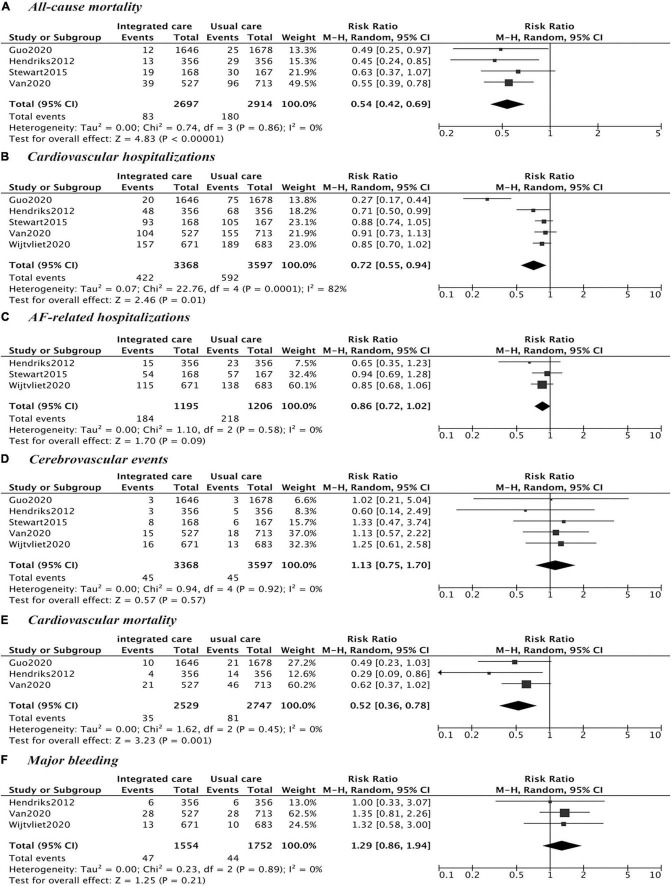
Comparison of integrated care and usual care. **(A)** All-cause mortality; **(B)** cardiovascular hospitalizations; **(C)** AF-related hospitalizations; **(D)** cerebrovascular events; **(E)** cardiovascular mortality; **(F)** major bleeding.

In comparison with usual care, integrated care was significantly associated with a 46% (RR = 0.54, 95% CI = 0.42–0.69, *P*_–heterogeneity_ = 0.86, I^2^ = 0%) and a 28% (RR = 0.72, 95% CI = 0.55–0.94, *P*_–heterogeneity_ = 0.0001, I^2^ = 82%) reduced risk of all-cause mortality and cardiovascular hospitalizations, without any significant heterogeneity (Figure 2). The meta-analysis failed to show a statistically significant benefit with the available data in AF-related hospitalizations (RR = 0.86, 95% CI = 0.72–1.02, *P*_–heterogeneity_ = 0.58, I^2^ = 0%), cerebrovascular events (RR = 1.13, 95% CI = 0.75–1.70, *P*_–heterogeneity_ = 0.92, I^2^ = 0%), cardiovascular mortality (RR = 0.60, 95% CI = 0.33–1.11, *P*_–heterogeneity_ = 0.11, I^2^ = 50%), and major bleeding events (RR = 1.29, 95% CI = 0.86–1.94, *P*_–heterogeneity_ = 0.89, I^2^ = 0%), when comparing with usual care; similarly, the heterogeneity was not observed in each pooled analysis. The funnel plots did not reveal any evidence of obvious asymmetry for the distribution of studies with the outcome of all-cause mortality, cardiovascular hospitalizations, AF hospitalizations, stroke, cardiovascular mortality, and major bleeding, respectively (Supplementary Figures 2–7).

In sensitivity analysis, repeated analyses were implemented several times by excluding each study at a time, and no significant change was observed in four outcomes, except for cardiovascular mortality (Supplementary Figures 8–13). After excluding the study done by Wijtvliet et al. (8), a significant reduced risk of cardiovascular mortality (RR = 0.52, 95% CI = 0.36–0.78, *P*_–heterogeneity_ = 0.45, I^2^ = 0%) was shown by only pooling results from two studies in Netherland (7, 9) and one in China (10).

## Discussion

This systematic review and meta-analysis by synthesizing available randomized controlled trials indicate that integrated care compared with usual care can effectively reduce cardiovascular hospitalizations, all-cause mortality, and cardiovascular mortality among AF patients.

Our study included three newly published RCTs (8–10), which were in line with the essence of the ESC Guidelines 2020 (2), emphasizing on the role of patient involvement and family/caregiver involvement. Our results found that the application of integrated care in AF patients can significantly reduce the risk of all-cause mortality and cardiovascular hospitalizations, but had no superior effect on AF-related hospitalizations, cerebrovascular events, and bleeding events, which was consistent with Gallagher’s report (14). Additionally, after excluding a study (8) with large confidence intervals, our study observed a remarkable 47% reduced risk of cardiovascular mortality by pooling two trials in Netherland (7, 9) and one trial in China (10), and the heterogeneity was largely reduced, further demonstrating the beneficial effect of AF integrated care. The possible reason might be that the experience in implementing nurse-led integrated care was uneven among hospitals included in the RACE 4 study (8) and then led to a wider interval confidence. In addition, ABC-adherent management is a simplified integrated care with especially concentration on three key elements of avoid stroke, better symptom management, and cardiovascular and comorbidity risk reduction. A meta-analysis (15) by pooling results from five observational studies, two studies with *post hoc* analysis, and one RCT also displayed the that ABC-adherent management have protective effect on all-cause mortality and cardiovascular mortality, further indicating that our results was robust.

Noteworthy, there was an apparent disconnection of the strong benefit observed (overall mortality, cardiovascular mortality, and cardiovascular hospitalizations) with the outcomes that the integrated care model is directly trying to improve (AF-related hospitalizations, cerebrovascular events, bleeding). One possible explanation for this discrepancy may be that the benefit of the integrated care model has more to do with increased overall contacts with the medical team. Another reason may be attributed to the multidisciplinary team approach and psychosocial management, which played a vital role in improving physical function, thus alleviating disease states, and finally reducing the risk among patients. The patients with AF were mostly over 50 years old and more commonly suffered from chronic disease or comorbidities. By using a multidisciplinary team with structured follow-up and clear communication, not only AF but also clinical deterioration or complications can be easily recognized. Besides, available evidence also indicated that simple cardiac risk factor management, such as diabetes and blood pressure management, can contribute to fewer all-cause deaths and cardiovascular hospitalizations (16–18), further indicating that integrated care could benefit the prognosis of AF patients. However, among the interventions of integrated care for AF patients, which interventions contributed the most to reducing the mortality of patients and other events remains to be confirmed with more evidence.

Although the intervention strategies varied among the included studies, they all emphasized the superiority of team-based integrated care approaches as shown in the Supplementary Table 3. Besides, the heterogeneity was not noticeable in each pooled analysis, which demonstrated the robust and stable results and would be beneficial for the conclusion to be drawn. For the detailed elements of each strategy across the five included studies (Supplementary Table 4), all considered four core elements including optimized stroke prevention, symptom control with rate or rhythm control, patient education/self-management, structured follow-up, and clear communication between primary and secondary care. In comparison with SAFETY study (6), the study by Hendriks et al. (7) added two more elements of psychosocial management and multidisciplinary team approach to their integrated management and observed lower risk of all-cause mortality among AF patients. In comparison with the ALL-IN trial (9), the mAFA II trial (10) added three more elements of management of cardiovascular risk factors/comorbidities, healthcare professional education, and strategies to promote medication adherence to its integrated care strategy and observed a lower risk of cardiovascular hospitalizations among AF patients. These may indicate that the more elements being considered, the more benefits patients would gain.

The focus of integrated AF management needs to utilize available resources to reduce stroke, improve symptoms, and treat comorbidities. Usual AF management often ignored the patient’s wishes, or the patient would subconsciously obey the doctor without expressing their preferences (19–21). Many studies (20–22) have shown that patient participation and joint decision-making were indispensable parts of the success of AF management. The integrated AF management in the latest guidelines (2) advocated strengthening patient education, making patients pay attention to stroke prevention and rhythm control, and fully understand their respective risks of death, stroke, and major bleeding, as well as their treatment burden. Future intervention strategies should include the involvement of the patient or family to stimulate the patient’s ability to self-manage their disease.

This study has some strengths. The effectiveness of integrated care was evaluated by comparing usual care, providing high-level evidence for the efficacy of integrated care management in the treatment of patients with AF. This study only included randomized control trials, which can help to reduce the heterogeneity. The meta-analysis by synthesizing available data demonstrated the general effect of integrated care in the endpoint such as all-cause mortality, cardiovascular hospitalizations, AF-related hospitalizations, cerebrovascular events, cardiovascular mortality, and major bleeding.

However, several limitations need to be noted. Firstly, although we know that integrated care is associated with patient outcomes, there is insufficient evidence to analyze patient-reported outcomes and health quality of life. Secondly, at present, there is no unified definition of integrated care for AF. The latest guidelines (2) suggested integrated AF care as an approach to AF management, which would change over time; it also emphasized the importance of patient involvement and shared decision-making. These make it more difficult to define patient interventions for integrated AF care. However, integrated care is a patient-centered, multidisciplinary-coordinated intervention strategy and is beneficial to patients, and should be widely recommended. Thirdly, treatment options may vary from study to study. For example, the study by Hendricks et al. was conducted in 2007–2008 (7), and hence likely all patient were on Vitamin K Antagonists (VKAs). Stewart et al. had more than 50% of their patients on VKAs (6). This could be a major confounder as currently the standard of care is direct oral anticoagulants (DOACs) and likely a big part of the results is going to be affected by a higher TTR in the integrated arm group. Fourthly, only five studies were selected and the elements of integrated care approach varied across the studies. This limitation might influence the generalizability. Hence, more studies in different countries are needed.

## Conclusion

This systematic review and meta-analysis with limited evidence showed that integrated care can reduce the risk of all-cause mortality, cardiovascular mortality, and cardiovascular hospitalizations in patients with AF compared with usual care. In other aspects, including AF-related hospitalizations, cerebrovascular events, and major bleeding, the integrated AF management performance was comparable with usual AF management.

## Author Contributions

XL conceived and designed the study. YL and WZ searched the data. YL analyzed the data. YL and XL drafted the manuscript. XL and JH supervised the study. PH, JH, HD, WZ, JL, and XL reviewed and interpreted the findings. All co-authors provided comments and approved the final version.

## Conflict of Interest

The authors declare that the research was conducted in the absence of any commercial or financial relationships that could be construed as a potential conflict of interest. The handling editor WZ declared a shared parent affiliation with the authors YL, PH, and JL at the time of review.

## Publisher’s Note

All claims expressed in this article are solely those of the authors and do not necessarily represent those of their affiliated organizations, or those of the publisher, the editors and the reviewers. Any product that may be evaluated in this article, or claim that may be made by its manufacturer, is not guaranteed or endorsed by the publisher.
