# Supplementary material for: Effect of Integrated Care on Patients With Atrial Fibrillation: A Systematic Review of Randomized Controlled Trials
Source: Front Cardiovasc Med. 2022 May 17;9:904090. doi: 10.3389/fcvm.2022.904090 (PMC9152009; doi:10.3389/fcvm.2022.904090)
Supplement: Supplementary file 1 [file Data_Sheet_1.docx]

**Supplementary Material**

Supplementary Material Table 1. Search strategy

Supplementary Material Table 2. The reason for the exclusion of publications

Supplementary Material Fable 3. The strategy applied to the intervention group and the control group

Supplementary Material Table 4. The essential elements of integrated atrial fibrillation management strategy adopted in each study

Supplementary Material Figure 1. Assessment of risk of bias in included studies

Supplementary Material Figure 2. Funnel plot of the effect of integrated care on all-cause mortality

Supplementary Material Figure 3. Funnel plot of the effect of integrated care on cardiovascular hospitalizations

Supplementary Material Figure 4. Funnel plot of the effect of integrated care on AF hospitalizations

Supplementary Material Figure 5. Funnel plot of the effect of integrated care on stroke

Supplementary Material Figure 6. Funnel plot of the effect of integrated care on cardiovascular mortality

Supplementary Material Figure 7. Funnel plot of the effect of integrated care on major bleeding

Supplementary Material Figure 8. Sensitivity analysis on the effect of integrated care on all-cause mortality

Supplementary Material Figure 9. Sensitivity analysis on the effect of integrated care on cardiovascular hospitalizations

Supplementary Material Figure 10. Sensitivity analysis on the effect of integrated care AF hospitalizations

Supplementary Material Figure 11. Sensitivity analysis on the effect of integrated care on stroke

Supplementary Material Figure 12. Sensitivity analysis on the effect of integrated care on cardiovascular mortality

Supplementary Material Figure 13. Sensitivity analysis on the effect of integrated care on major bleeding

Supplementary Material Table 1. Search strategy

| PubMed | | |
| --- | --- | --- |
| 1 | "Atrial Fibrillation" |  |
| 2 | delivery of health care, integrated [MeSH Terms] OR "integrated health care" OR "integrated care" OR nurse-led care OR "Interdisciplinary Communication" OR "Interdisciplinary Communications" OR multidisciplinary OR "outpatient" OR "ambulatory care" OR nursing OR "ABC pathway" OR "ABC Care" |  |
| 3 | "all-cause mortality" OR "mortality" OR "death" OR "hospitalization" OR "hospitalizations" OR "hospital admissions" OR "stroke" OR "major bleeding" OR "adherence to guidelines" OR "quality and outcomes" OR "Multimorbidity" OR "Anticoagulation" |  |
| 4 | ((#1) AND #2) AND #3 |  |
| Embase | | |
| 1 | 'Atrial fibrillation' |  |
| 2 | 'Integrated health care system' OR 'nurse-led' OR 'interdisciplinary communication' OR 'multidisciplinary team' OR outpatient OR 'ambulatory care' OR 'ABC pathway' ' OR 'ABC Care' |  |
| 3 | 'all-cause mortality' OR mortality OR death OR hospitalization OR 'hospital admission' OR 'cerebrovascular accident' OR bleeding OR 'patient compliance' |  |
| 4 | #1 AND #2 AND #3 |  |
| Web of Science | | |
| 1 | TS= (Atrial Fibrillation) |  |
| 2 | ALL=(delivery of health care OR integrated care OR nurse-led care OR integrated health care OR ABC pathway OR ABC care) |  |
| 3 | (#1) AND #2 |  |

Supplementary Material Table 2. The reason for the exclusion of publications

| **Author** | **Year** | **Reason for exclusion** |
| --- | --- | --- |
| Stewart et.al. | 2016 | No focus on AF |
| Cater et.al. | 2016 | Not randomized controlled trial |
| Andrikopoulos et.al. | 2014 | No control group |
| Giugliano et.al. | 2014 | No focus on integrated care |
| Hendriks et.al. | 2013 | Duplicate data |
| Hendriks et.al. | 2014 | Duplicate data |
| Hendriks et.al. | 2019 | Duplicate data |
| Proietti et.al. | 2018 | No focus on integrated care |
| Proietti et.al. | 2018 | Not randomized controlled trial |
| Ariyarathna et.al. | 2019 | No control group |
| Pastori et.al. | 2019 | Not randomized controlled trial |
| Domek et.al. | 2020 | No focus on AF |
| Proietti et.al. | 2020 | Not randomized controlled trial |
| Yang et.al. | 2020 | Not randomized controlled trial |
| Proietti et.al. | 2021 | Not randomized controlled trial |
| Gumprecht et.al. | 2020 | Not randomized controlled trial |
| Kozieł et.al. | 2020 | Not randomized controlled trial |

Supplementary Material Table 3. The strategy applied to the intervention group and the control group

| **Author，Publication year,**  **Country,**  **Study design** | **Intervention group** | **Control group** |
| --- | --- | --- |
| Stewart et al, 2015,  Australia, pragmatic multicenter, randomized controlled trial | **Intervention group (The SAFETY group)**   - Proactive management with respect to optimization of gold-standard drug treatment and non-pharmacological management - In-hospital assessment to establish potential barriers to post-discharge management and initial contact to develop a therapeutic relationship with the patient and their family or career - Structured post-discharge care, consisting of a home visit 7–14 days after discharge followed by a combination of repeat home visits, scheduled clinic reviews, and telephone follow-up - Key components specifically tailored to the management of chronic atrial fibrillation and any substantial comorbid disorders: advanced clinical assessment (including ECG Holter monitoring); review of home and social environment; education about symptoms; review of drugs and treatment plan relative to gold standards; identification of any barriers to follow-up attendance and optimum self-care; management of other issues identified at discharge in the care plan; documentation of any actions taken, including communications with other health-care providers; adjustment or prescription of drugs or treatments; referral to primary care, and urgent or emergency care if needed; referral to other relevant community services (including exercise programs, social work support, and community pharmacists); and acting as a resource at any time via patient-initiated contact (telephone) | **Control group (The standard management group)**   - Ad-hoc management as per usual standards of clinical care (subsidized access to routine medical care, hospital care, and pharmacotherapy) |
| Hendrisks et al, 2012,  Netherland,  One-center Randomized clinical trial | **Intervention group (Nurse-led care group)**   - Based on the chronic care model, it consisted of nurse-led outpatient care steered by decision support software based on the guidelines and supervised by a cardiologist. - Before the first visit, patients underwent laboratory testing, electrocardiogram, Holter monitoring and echocardiography. - At the first visit, a nurse specialist took the patient's history and informed them about the pathophysiology of AF, its symptoms and possible complications, the results of the diagnostic tests and treatment options - The dedicated software was used to guide comprehensive management of AF and associated cardiovascular conditions. - Patients were instructed about rate and rhythm control as well as prophylactic vascular therapy (including strict anticoagulation monitoring), and about when to report to the hospital. - Visits to the nurse were scheduled to last 30 min. Follow-up visits were planned at 3, 6, and 12 months, and every 6 months thereafter. Patients could contact the nurse in person or by telephone between planned visits as needed. During follow-up visits, psychosocial support and educational interventions were repeated. | **Control group (Usual care group)**   - Usual care was provided to patients in the control group by a cardiologist in the outpatient clinic during visits scheduled to last 20 min for the first visit and 10 min for follow-up visits. |
| Wijtvliet et al, 2020,  Netherlands,  Multi-center Randomized clinical trial | **Intervention group (Nurse-led care group)**   - Treatment of patients by a specialized nurse using guidelines-based decision-support software ensuring comprehensive treatment of atrial fibrillation and associated conditions, covering cardiovascular risk factor management, antithrombotic treatment, rate control, and rhythm control. - Complete cardiological diagnostic tests and treatments were installed during the first outpatient visit. All test results were extensively discussed, and treatments adapted as needed and confirmed onsite with the supervising cardiologist, all during the first visit. - The nurse provided psychosocial support as well as personalized education on pathophysiology, symptoms, and complications of atrial fibrillation to enhance adherence of patients. | **Control group (Usual care group)**   - Usual care consisted of routine outpatient management by a cardiologist without a specified clinical pathway. |
| Van et al,  2020,  Netherlands,  Cluster randomized pragmatic non-inferiority trial | **Intervention group (Integrated care)**   - Quarterly follow-up visits, included special attention to detection of signs of heart failure, adequate rate control, evaluation of the need for laboratory testing or ECG, lifestyle improvement and patient education/empowerment - Tailored anticoagulation monitoring: In VKA patients: regular INR measurements in the primary care practice or if necessary, at home - Cardiologists and anticoagulation clinics were easily accessible for consultation | **Control group (Usual care)**   - Variable and delivered by different health care professionals (cardiologists, specialized AF nurses, anticoagulation clinics, general practitioners, practice nurses), without one designated coordinator. Some patients did not receive any care during follow-up. In VKA patients, INR measurements were performed by anticoagulation clinics by venipuncture. Some patients measured the INR themselves at home. |
| Guo et al,  2020,  China,  Cluster randomized controlled trial | **Intervention group (Integrated care)**   - The doctors used the mAFA platform to manage patients with AF, which provided clinical decision support tools to facilitate guideline-based treatment recommendations, educational materials and patient involvement strategies with self-care protocols, and structured follow-up, to support implementation of the ABC pathway for integrated or holistic AF management. | **Control group (Usual care)**   - Patients in the usual care group received treatment and management by local doctors according to local clinical practice. |

Supplementary Material Table 4. The essential elements of integrated atrial fibrillation management strategy adopted in each study

| **Intervention strategy*** | **Stewart et al, 2015** | **Hendriks et al, 2012** | **Wijtvliet et al, 2020** | **Van et al, 2020** | **Guo et al,**  **2020** |
| --- | --- | --- | --- | --- | --- |
| Optimized stroke prevention | **●** | **●** | **●** | **●** | **●** |
| Symptom control with rate or rhythm control | **●** | **●** | **●** | **●** | **●** |
| Management of cardiovascular risk factors/comorbidities | **●** |  | **●** |  | **●** |
| Patient education/self-management | **●** | **●** | **●** | **●** | **●** |
| Healthcare professional education |  |  | **●** |  | **●** |
| Lifestyle modification |  |  |  | **●** |  |
| Psychosocial management |  | **●** | **●** |  |  |
| Strategies to promote medication adherence | **●** |  | **●** |  | **●** |
| Multidisciplinary team approach |  | **●** | **●** | **●** | **●** |
| Structured follow-up and clear communication between primary and secondary care | **●** | **●** | **●** | **●** | **●** |

^*^The integrated atrial fibrillation management strategy suggested by 2020 ESC guidelines [reference: Hindricks G, et al: 2020 ESC Guidelines for the diagnosis and management of atrial fibrillation developed in collaboration with the European Association for Cardio-Thoracic Surgery (EACTS): The Task Force for the diagnosis and management of atrial fibrillation of the European Society of Cardiology (ESC) Developed with the special contribution of the European Heart Rhythm Association (EHRA) of the ESC. Eur Heart J 2021, 42(5):373-498.]


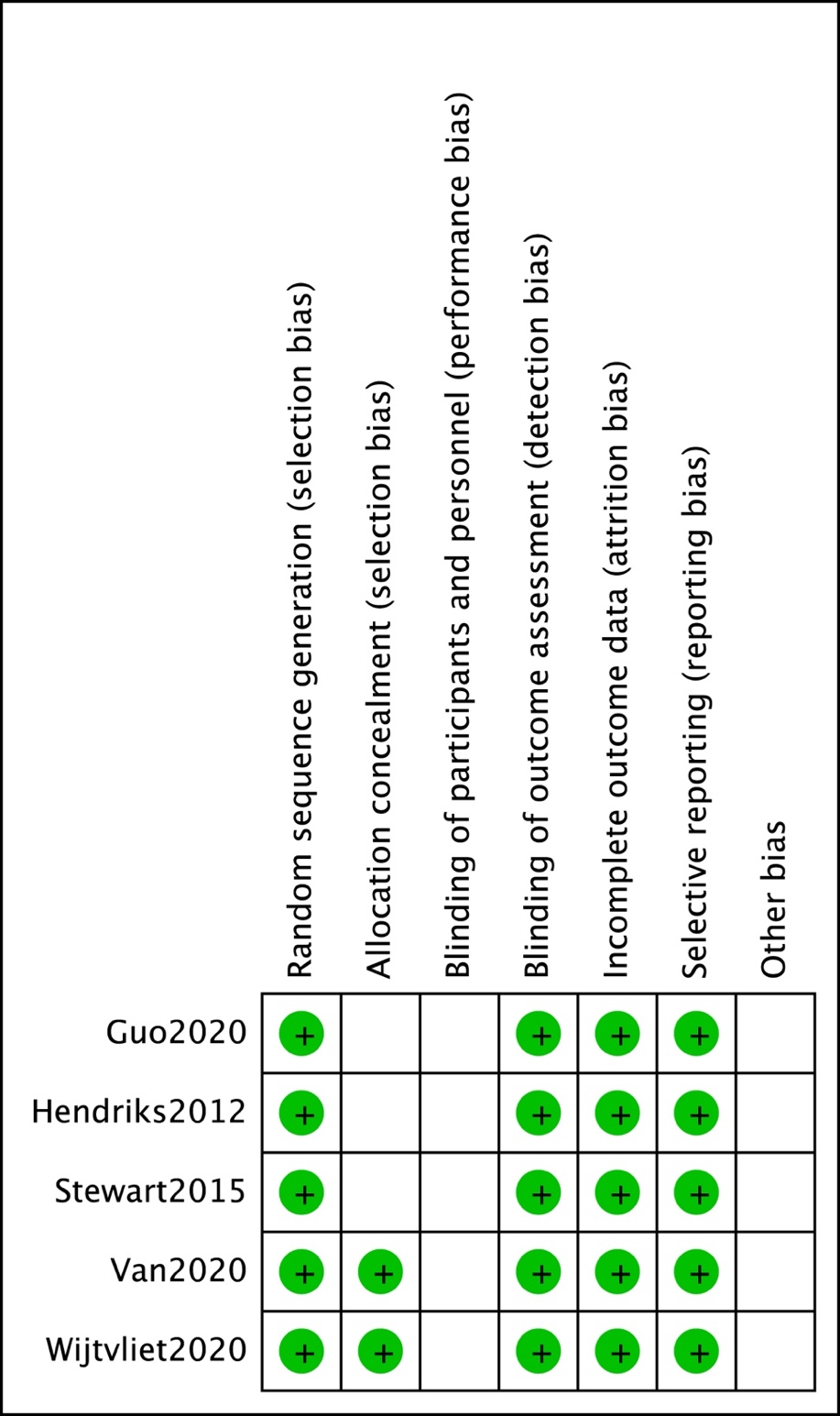


Supplementary Material Figure 1. Assessment of risk of bias in included studies


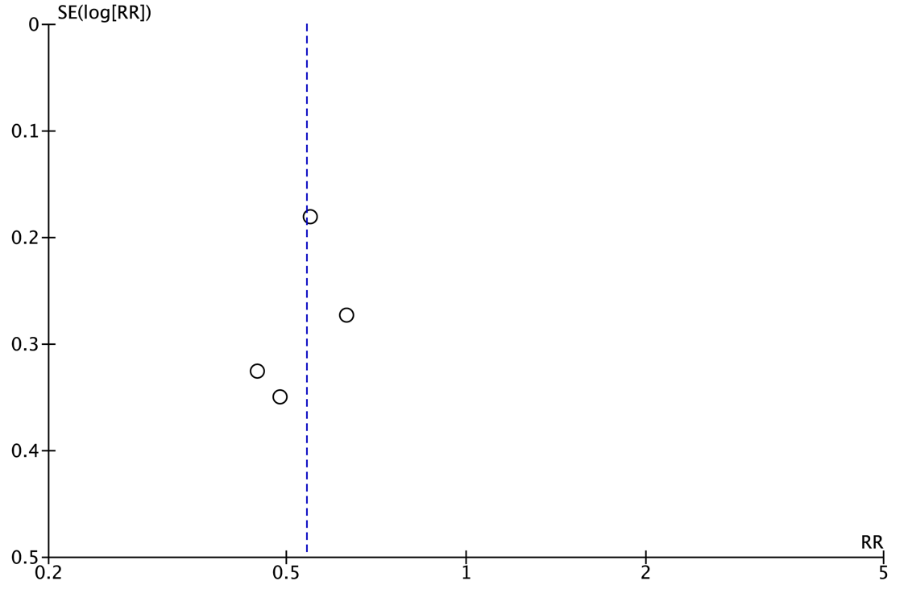


Supplementary Material Figure 2. Funnel plot of the effect of integrated care on all-cause mortality


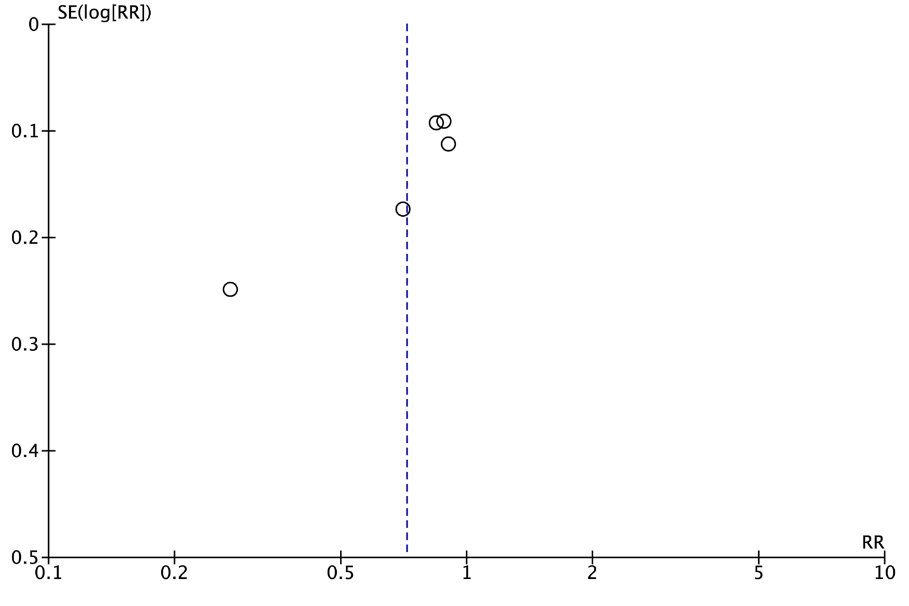


Supplementary Material Figure 3. Funnel plot of the effect of integrated care on cardiovascular hospitalizations


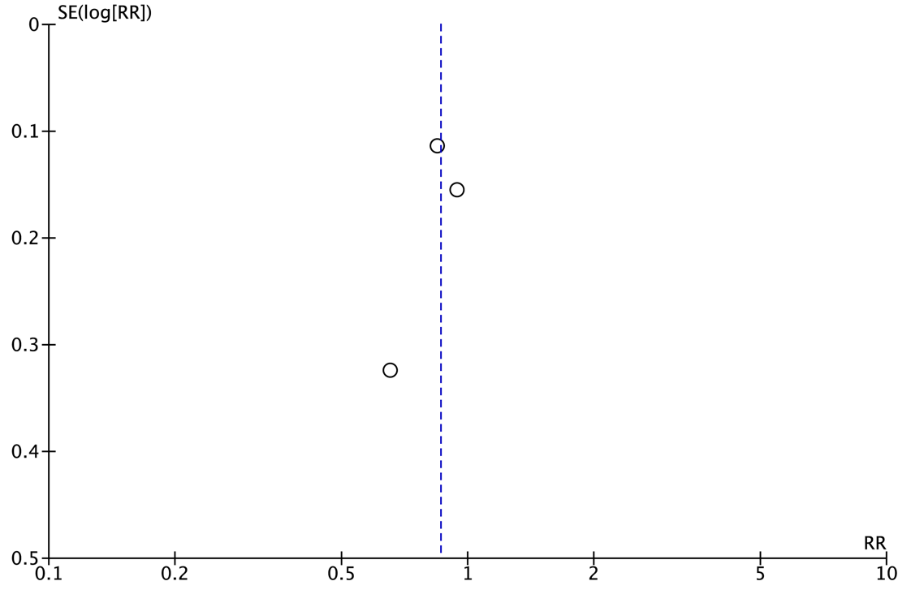


Supplementary Material Figure 4. Funnel plot of the effect of integrated care on AF hospitalizations


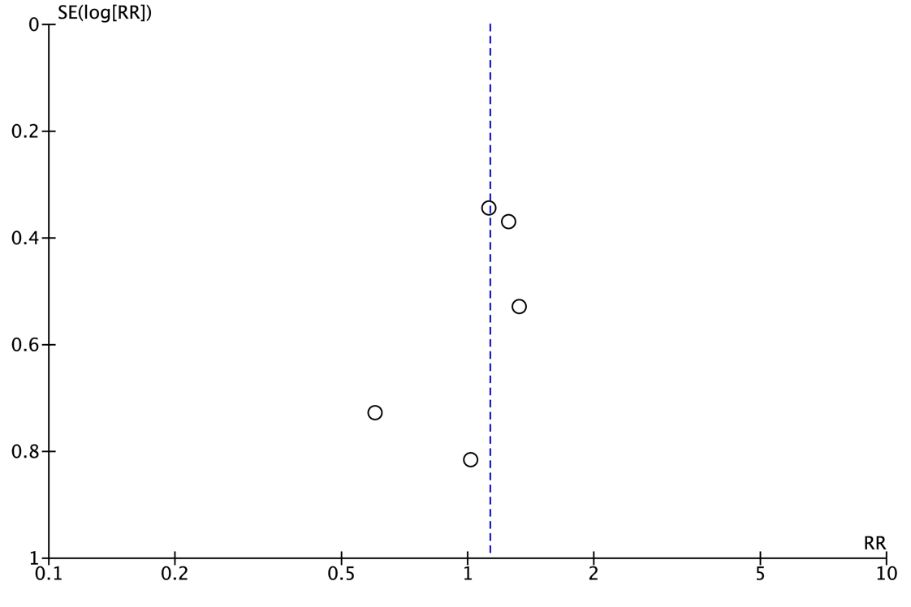


Supplementary Material Figure 5. Funnel plot of the effect of integrated care on stroke


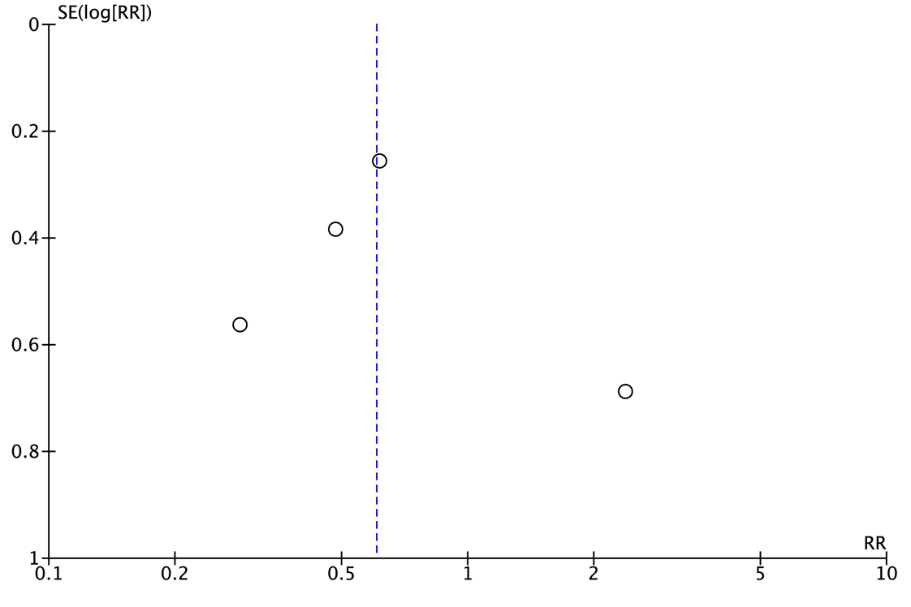


Supplementary Material Figure 6. Funnel plot of the effect of integrated care on cardiovascular mortality


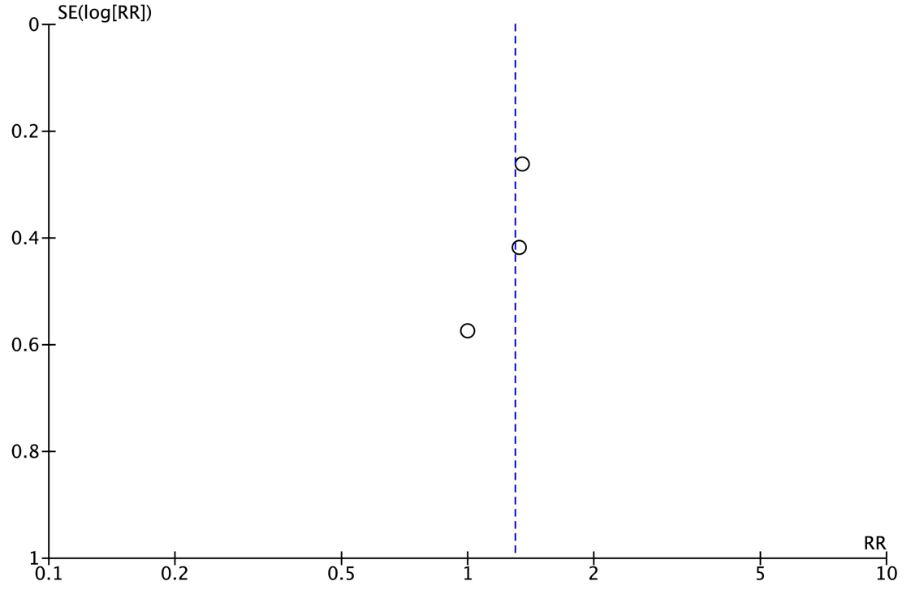


Supplementary Material Figure 7. Funnel plot of the effect of integrated care on major bleeding


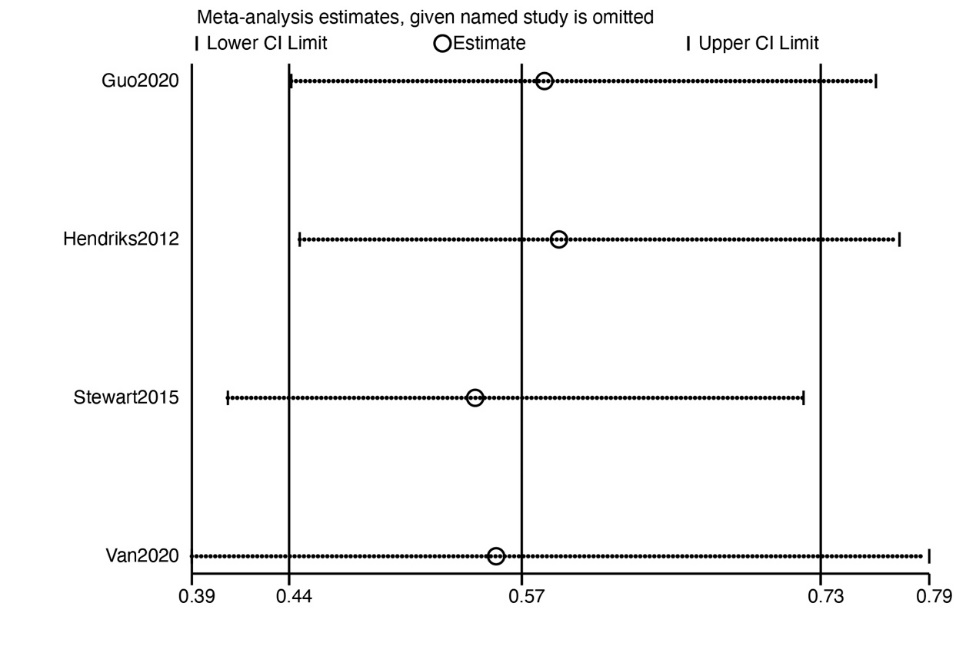


Supplementary Material Figure 8. Sensitivity analysis on the effect of integrated care on all-cause mortality


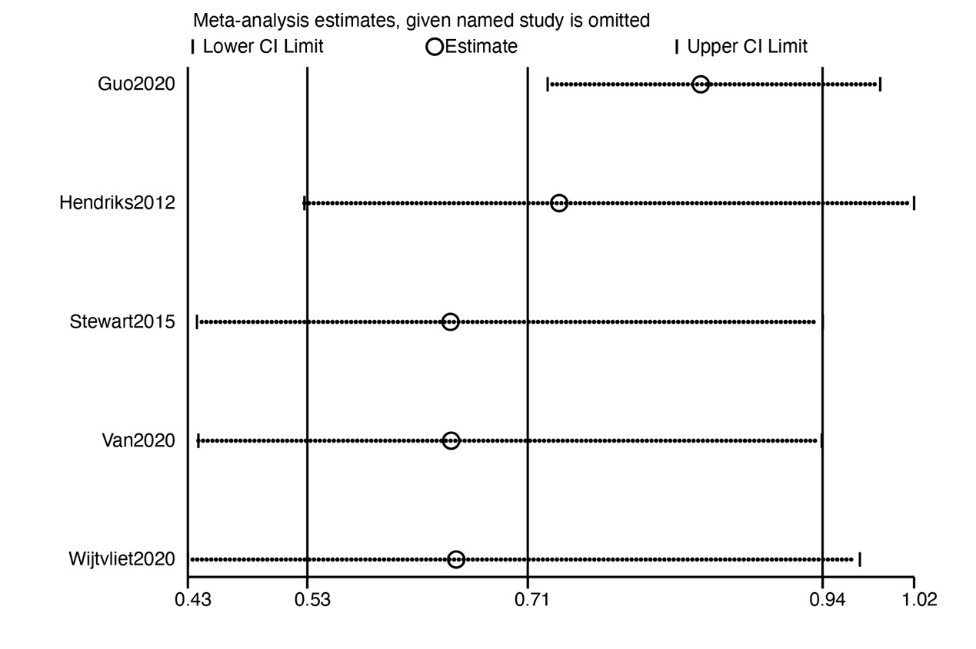


Supplementary Material Figure 9. Sensitivity analysis on the effect of integrated care on cardiovascular hospitalizations


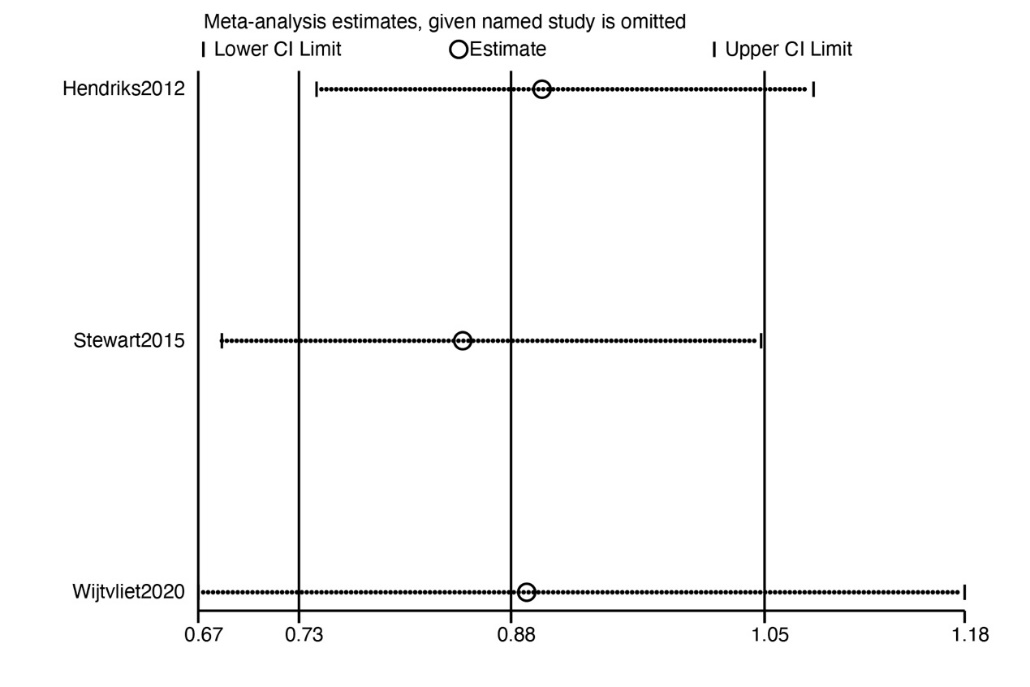


Supplementary Material Figure 10. Sensitivity analysis on the effect of integrated care AF hospitalizations


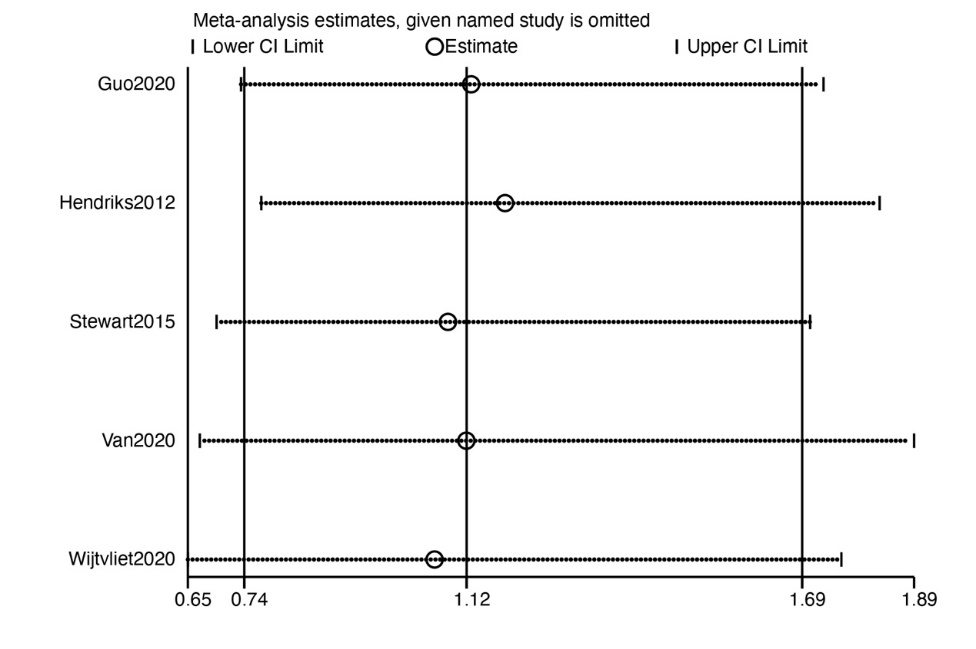


Supplementary Material Figure 11. Sensitivity analysis on the effect of integrated care on stroke


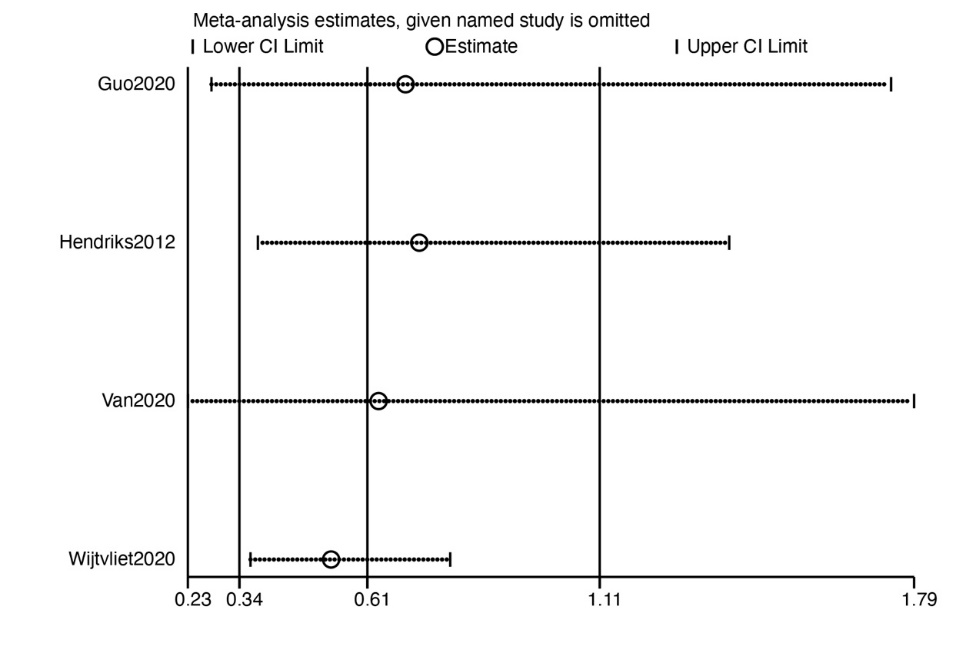


Supplementary Material Figure 12. Sensitivity analysis on the effect of integrated care on cardiovascular mortality


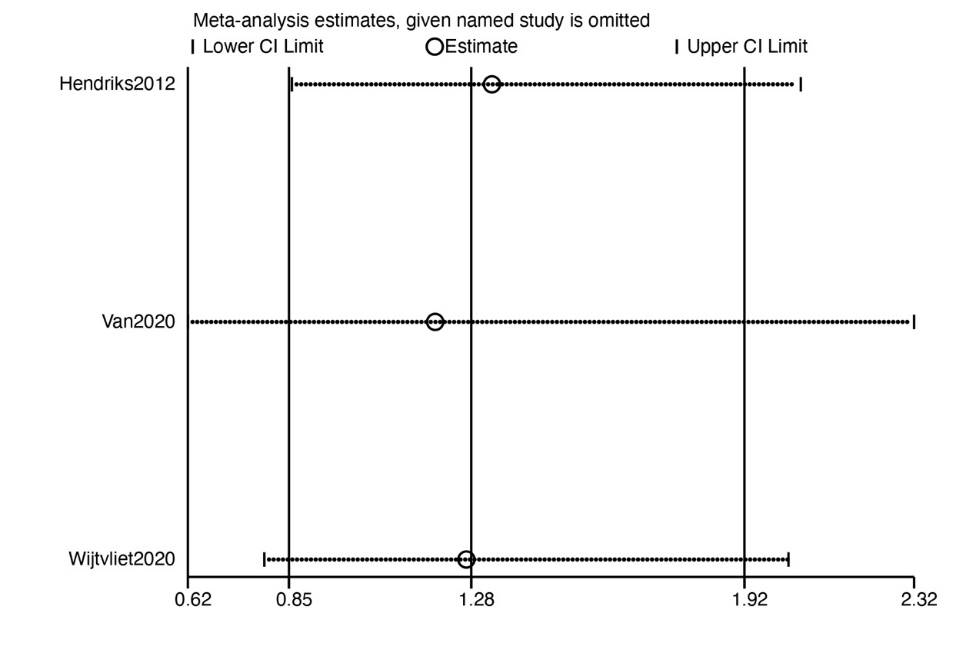


Supplementary Material Figure 13. Sensitivity analysis on the effect of integrated care on major bleeding
